# Efficacy of an online attachment priming and mindfulness practice intervention for insomnia, stress, and emotion regulation in university students: A randomized controlled trial

**DOI:** 10.1111/aphw.70202

**Published:** 2026-08-05

**Authors:** Jodie C. Stevenson, Meghann Matthews, Umair Akram

**Affiliations:** ^1^ Independent Researcher Lincoln UK; ^2^ School of Psychology University of Sheffield Sheffield UK; ^3^ School of Psychology University of Lincoln Lincoln

**Keywords:** attachment priming, emotion regulation, insomnia, mindfulness, stress, student mental health

## Abstract

Despite the availability of student well‐being services, university students remain highly susceptible to mental health challenges. Recent research highlights the potential of mindfulness‐based interventions and attachment security priming in enhancing psychological well‐being. This study compared the immediate and sustained effects of digitally delivered attachment priming and mindfulness‐based practices on student mental health over one week. Using a randomized controlled design, 122 UK undergraduate students were assigned to one of three groups: repeated attachment security priming, mindfulness practice, or a waitlist control. Participants completed baseline assessments of stress, insomnia, attachment anxiety, emotion regulation, and mindfulness. The intervention groups engaged in five consecutive days of digital practice, and all participants completed follow‐up assessments immediately after (T2), one week later (T3), and four weeks later (T4). Compared with the control group, both intervention groups showed significant long‐term improvements in stress, insomnia, emotion regulation, and academic dropout intention. Attachment priming produced the most consistent and substantial benefits across time points. Each intervention also resulted in specific gains in mindfulness and attachment‐related outcomes. These findings support the integration of digital attachment priming and mindfulness‐based programs as promising supplements or alternatives to traditional university mental health services, especially when timely, accessible support is lacking.

## INTRODUCTION

The progression to university level study is commonly associated with significant academic and psychosocial challenges that leave university students vulnerable to psychological distress and disturbed sleep and difficulties in emotion regulation (Akram et al., 2020; Taylor et al., 2013). Indeed, university study typically involves a less structured learning environment that emphasizes autonomy of learning paired with increased academic expectations in relation to quality of work and general capability (Cleary et al., 2010). Moreover, because of increasingly higher costs of living and increased tuition fees, financial strain associated with living on a limited self‐managed budget for the first time may be particularly difficult for UK students (O'Neill et al., 2018). By contrast, in the absence of parental supervision, social and collegial activities related to the university lifestyle present opportunities for increased alcohol intake, potential substance use, and formation of sexual relationships (Cleary et al., 2010; Foster et al., 2023). These factors possibly create an environment that precipitates mental health difficulties among vulnerable young adults.

Despite a pattern of yearly decline in student mental health (Gardani et al., 2022), institutional well‐being services consistently fail to meet the increasing demand for support (Cleary et al., 2010; Thorley, 2017). Crucially, several reports evidence institutional processes to be arduous and often ineffective, particularly when approaching common psychiatric conditions (Hardre & Reeve, 2003; Harrod et al., 2014; O'Neill et al., 2018). Therefore, it is vital for student well‐being services to expand the respective programs offered alongside the scope of symptoms covered (Akram et al., 2020). Here, we examine the role of two potentially therapeutic approaches that may serve to improve mental health outcomes in the student population: repeated mindfulness‐based practice (MBP) and attachment security priming.

## MINDFULNESS BASED PRACTICE

The methodological principles of Buddhist mindfulness meditation have long been incorporated into Western therapeutic protocols for psychological and physical well‐being (Ryan et al., 2021). Mindfulness‐based theories state that trait mindfulness enhances one's capacity to experience thoughts, feelings, and present moment experience with an attitude of nonjudgment and acceptance (Brown & Ryan, 2003). The capacity to be mindful is considered an enduring and multifaceted trait‐like individual difference (i.e. dispositional mindfulness; Brown & Ryan, 2003), often associated with improved psychological well‐being and reduced reports of stress, anxiety, and depression (Weinstein et al., 2009). Indeed, mindfulness practice (MBP) and mindfulness‐based interventions deployed across a broad spectrum of populations yield clinically significant improvements for those experiencing psychological distress (Luoma & Villatte, 2012), psychiatric distress (Creswell, 2017), poor sleep (Winbush et al., 2007), and medical illness (Gu et al., 2015; Ludwig, 2008). Here, deploying meditation practices for longer periods is known to moderate the efficacy of MBP in relation to increased mindfulness and psychological well‐being and reduced psychological distress (Carmody & Baer, 2007).

In the current context, research surrounding the recent incorporation of MBP into student well‐being programs has revealed promising outcomes in relation to the experience of stress and anxiety (see Gu et al., 2015, for review). Given the wider efficacy of MBP, these outcomes may be extrapolated to further sources of student distress. However, although a consistent pattern of improved mental health outcomes is observed in relation to both dispositional mindfulness and interventional benefits of MBP, mechanisms underlying the emergence of dispositional mindfulness independent of contemplative practice remain elusive (Gu et al., 2015). Given that attachment anxiety (i.e. fear of abandonment) and avoidance (i.e. avoiding intimacy; Bamber & Kraenzle Schneider, 2016; Bartholomew & Horowitz, 1991; Brennan et al., 1998) often predict aspects of dispositional mindfulness, exploring the role of adult attachment may offer further insight into additional methods for promoting student mental health and well‐being (Stevenson et al., 2019, 2021).

### Attachment security priming

Attachment security priming has been used to understand the various cognitive processes that pertain to attachment‐related affect and behavior (Canterberry & Gillath, 2013; Gillath et al., 2016; Gillath & Karantzas, 2019). Security priming involves repeated exposure to stimuli designed to elicit a sense of comfort, safety, and love, aiming to improve the accessibility and salience of mental representations of memory (Gillath et al., 2016; Gillath & Karantzas, 2019). In turn, the neural diffusion of semantic and affective node activation triggers a sense of security akin to the feeling provoked by the love and comfort of another supportive individual, known as an attachment figure (Gillath & Karantzas, 2019). Following this, the relative roles of individual attachment styles (i.e. anxiety or avoidance) proposed by the two‐dimensional model should be further explored (Shaver & Mikulincer, 2008).

Attachment anxiety emerges in response to inconsistent care, precipitating hyperarousal of the attachment system and emerging negative cognitive processes (i.e. worry and rumination) related to abandonment and rejection. Eventually, these processes perpetuate an increased behavioral effort to seek proximity and protection (Shaver & Mikulincer, 2008). In contrast, as a diminution of the attachment system, avoidance stems from the experience of rejecting or nonresponsive caregiving. Here, individuals become in denial of their attachment needs, actively suppressing vulnerability and proximity seeking (Mikulincer & Shaver, 2003). Cross‐sectional approaches to security priming have been found to improve psychological well‐being (McGuire et al., 2018; Mikulincer & Shaver, 2003), increase compassion and altruism (Gilbert, 2005; Mikulincer et al., 2005), and reduce aggression (Mikulincer & Shaver, 2001). Although longitudinal approaches remain sparse, repeated attachment priming (over one to several weeks) and cumulative exposure are associated with improvements in psychological well‐being, self‐perception, and compassion for others (Carnelley & Rowe, 2010; Gillath et al., 2008; McGuire et al., 2018).

Although some changes can be fleeting, others have been documented as lasting much longer. The literature consistently evidences both short‐ and long‐term benefits of attachment security priming (Gillath et al., 2008). Throughout the literature, studies have detailed the salutary effects of experimentally activating one's secure close relationship schemas (priming). Priming studies have documented that enhancing attachment security, or insecurity, produces a range of style‐congruent behaviors (Gillath et al., 2008; Mikulincer & Shaver, 2001). Although our attachment style is amenable to change in relation to brief moments and situations, this is only within a range determined by our stable, dispositional attachment orientation (Gillath et al., 2009). Research has largely focused on the role of mindfulness in predicting psychological well‐being, particularly within intervention paradigms and personality frameworks (Gu et al., 2015), with comparatively limited attention given to the developmental origins of dispositional mindfulness. Drawing on attachment theory, however, emerging work positions adult attachment orientation as a key antecedent of mindfulness rather than a reciprocal correlate. Attachment theory posits that early relational experiences give rise to relatively stable internal working models that organise attention, emotion regulation, and responses to internal experience (Bowlby, 1969, 1973). These regulatory patterns map closely onto the core components of mindfulness, such that individuals high in attachment anxiety, characterized by hyperactivating strategies and cognitive preoccupation, are less able to sustain nonjudgmental present‐moment awareness, whereas those high in avoidance, marked by deactivating and suppressive strategies, tend to disengage from internal states, thereby constraining mindful awareness (Stevenson et al., 2021). In contrast, secure attachment facilitates greater affect tolerance and metacognitive capacity, which are theorized to underpin mindfulness.

Consistent with this theoretical account, researchers argue that attachment orientation functions as a primary developmental precursor to dispositional mindfulness rather than the relationship being bidirectional (Stevenson et al., 2021). Their empirical findings indicate that attachment insecurity predicts lower levels of mindfulness, supporting a unidirectional pathway in which attachment‐related regulatory strategies shape the emergence and stability of mindful awareness. This perspective aligns with broader developmental models suggesting that capacities for attention regulation and emotional awareness are scaffolded within early attachment relationships and subsequently generalize to intrapersonal processes, including mindfulness. The salience of emotion regulation within these frameworks is well established, given its robust associations with psychological distress and resilience across populations. Accordingly, interventions targeting both attachment‐related processes and mindfulness may exert synergistic effects on mental health outcomes through their shared influence on regulatory functioning (Caldwell et al., 2010; Stevenson et al., 2017).

### The present study

Both attachment security and mindfulness are related to therapeutic outcomes in higher education populations. Specifically, throughout the literature both attachment orientation and mindfulness have been associated with adaptive coping behaviors and the ability to adapt and persist in higher education, as well as increasing mental well‐being (Hatton‐Bowers et al., 2023; Nottage et al., 2022; Stevenson et al., 2017). Therefore, the current randomized controlled trial sought to explore the therapeutic potential of attachment priming and MBP in improving mental health outcomes (i.e. stress, insomnia, emotion regulation, and dropout intent) among university students.

Here, we chose to focus on improving prevalent, yet modifiable, difficulties pertaining to the experience of insomnia, stress, and emotion regulation. Indeed, recent data from a large sample of UK university students indicated that 84.5% of students presented moderate to significantly high levels of perceived stress, whereas approximately 55% of UK students were at risk for insomnia (Akram et al., 2023). With that in mind, increased academic performance has been positively associated with emotion regulation strategies and longer, more regular sleep (Okano et al., 2019; Romo et al., 2025).

In the present study, participants were randomized to one of three conditions: repeated security priming, mindfulness practice, or waitlist control. Following the completion of all questionnaires at baseline (T1), those allocated to the experimental treatment groups completed five consecutive days of either attachment security priming or MBP. Each group then completed the battery of questionnaires again: on the immediate completion of Day 5 (T2), 1 week after the final session (T3), and 4 weeks postcompletion (T4).

This trial was designed to assess the effects of two distinct approaches, attachment security priming and mindfulness practice, on key indicators of student mental health, and to determine whether their impact exceeded that of a nonactive control. The three‐arm design permitted both an evaluation of each intervention against usual conditions and a direct comparison of their relative benefits. Iterating upon that model, the primary aim was to establish whether brief, digitally delivered sessions of either approach produced measurable and sustained improvements in stress, sleep disturbance, emotion regulation, and intentions to withdraw from university. A secondary aim was to examine whether the two interventions influenced attachment‐related processes and dispositional mindfulness in different ways, thereby providing insight into their mechanisms of action. We anticipated that both interventions would outperform the waitlist control on primary mental health outcomes, and that attachment security priming would show broader effects on attachment dimensions. We also expected that gains in attachment security would precede and support increases in dispositional mindfulness. These aims and hypotheses define the comparative and mechanistic focus of the study and guide the interpretation of the findings.

## METHOD

### Ethical statement

**FIGURE 1:**
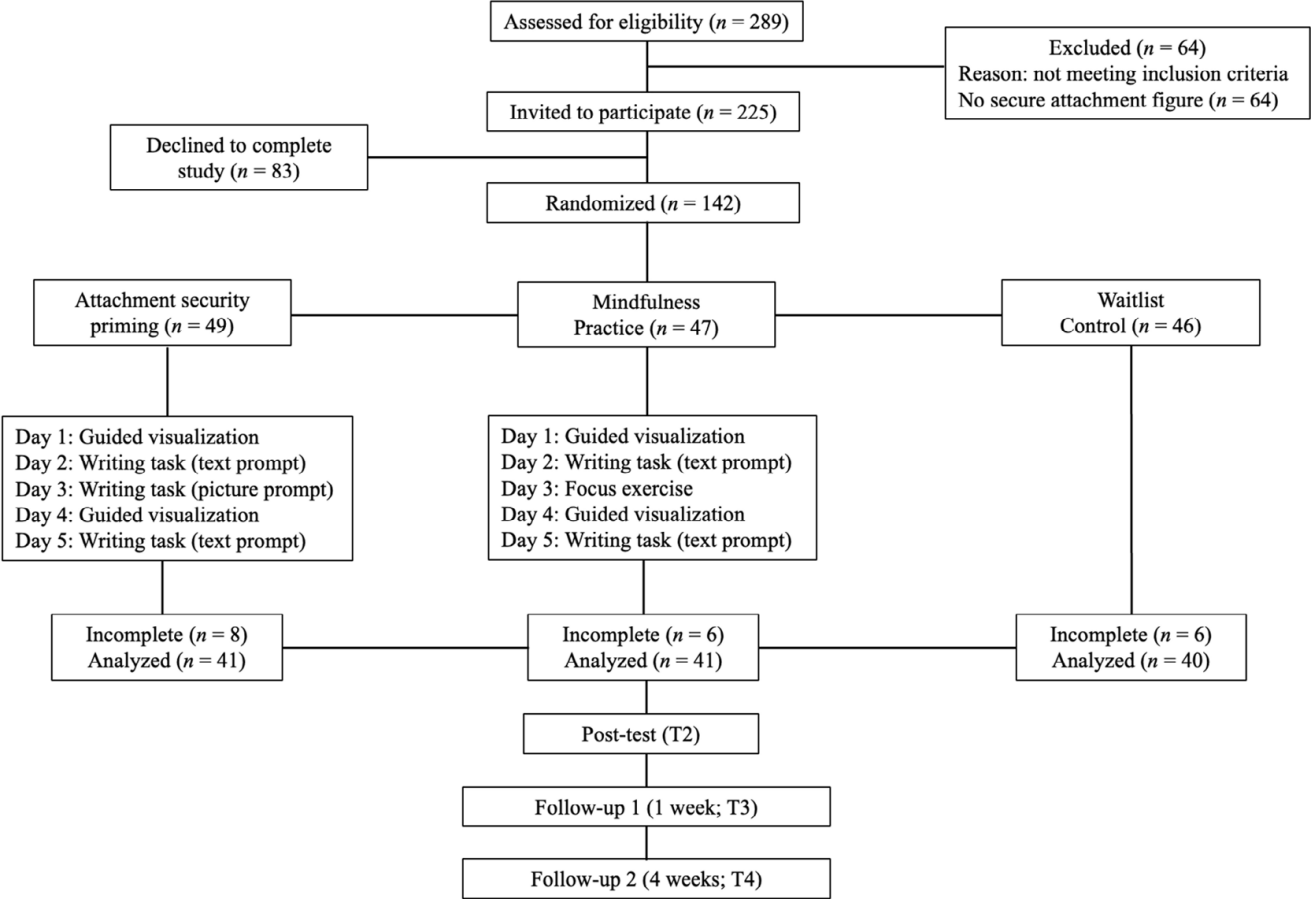
Participant and intervention flow diagram.

The authors assert that all procedures contributing to this work comply with the ethical standards of the relevant national and institutional committees on human experimentation and with the Helsinki Declaration of 1975, as revised in 2008. All procedures involving human subjects/patients were approved by the University of Lincoln's Research Ethics Committee (Project ID = 2934). This protocol was preregistered on the Open Science Framework database (DOI: DOI 10.17605/OSF.IO/VM9JC). Voluntary online informed consent was provided by all participants prior to data collection. The study flow chart of the randomized clinical trial is presented in Figure 1.

### Participants

Undergraduate students were recruited for course credit or monetary remuneration using a university‐wide email distribution list. The screening procedure consisted of an online questionnaire. Eligibility was based on the following requirements: current enrolment in an undergraduate course and confirmation of at least one secure attachment figure. Screening was completed by *N* = 206 students, where *n* = 64 failed to identify a secure attachment figure and were subsequently excluded. This left *N* = 142 students who were invited to take part in the trial. The final sample included *N* = 122 students who competed full protocol at all 4 time points (93.4% British, 88.5% female, *M*_age_ = 20.85, SD = 3.44, range = 18–45). Based on an a priori analysis, using G*Power (Faul, 2014), at least *N* = 114 were required to detect a medium effect (*f* = 0.25; *α* = 0.05, power = 0.85).

### Design and randomization

Simple randomization was conducted using an online research randomization tool (randomizer.org). Two randomization procedures were employed. First, each participant was assigned a unique study number (1–142; see Figure 1) to preserve anonymity. Second, participants were randomly assigned to one of three experimental conditions using simple randomization (codes 1–3). The allocation sequence was concealed from personnel involved in enrolment and data collection, minimizing selection and allocation bias. Through this process, participants were randomly allocated to one of three conditions: repeated attachment security priming, repeated mindfulness practice, or a waitlist control. Here, a 3 × 4 (condition × time) between‐subjects, repeated measures design compared the efficacy of repeated attachment security priming and repeated mindfulness practice in improving student mental health. The protocol included a posttest assessment and two follow‐up time points (1 week and 1 month).

### Measures

The Attachment Networks and Relationship‐specific Attachment Styles (ANRA) measure confirmed whether participants had at least one secure attachment figure (Rowe & Carnelley, 2003). Participants were presented with four descriptions representing different attachment prototypes (Hazan & Shaver, 2017) and indicated whether there was a close significant other in their life that matched each description (yes or no) and how representative each prototype was of how they feel in that relationship using a Likert scale from 1 (*not very representative*) to 5 (*very representative*). Participants who indicated having a secure attachment figure with a representative score of 3 and above were eligible to take part in the study. Secure attachment figures were measured globally and were not specific to romantic relationships.

Next, the self‐report Experiences in Close Relationship‐12 scale (ECR‐12; Lafontaine et al., 2016) was administered. The scale's 12 items are divided into two 6‐item subscales that represent the two underlying dimensions of adult attachment: anxiety and avoidance. Participants rated their feelings using a Likert scale ranging from 1 (*disagree strongly*) to 7 (*agree strongly*), with higher scores reflecting a greater endorsement of that construct. Cronbach's *α* for the present sample was .87 for anxiety and .89 for avoidance. Finally, the nine‐item self‐report Adult Disorganized Attachment scale (ADA) examined the level of adult disorganized attachment. Participants were asked to rate their agreement with each statement using a Likert scale ranging from 1 (*strongly disagree*) to 7 (*strongly agree*). The ADA has been shown to have high internal consistency (Cronbach's *α* = .91; Paetzold et al., 2015). The Cronbach's *α* for the current sample was .88.

The Five Facet Mindfulness Questionnaire Short Form (FFMQ‐ SF; Bohlmeijer et al., 2011) is a 24‐item self‐report measure of five subscales of dispositional mindfulness: acting with awareness (5 items), describing (5 items), observing (4 items), nonjudging (5 items), and nonreacting (5 items). Participants indicated the relatability of each item using a 5‐point Likert scale ranging from 1 (*never or very rarely true*) to 5 (*very often or always true*). In the present sample, the *α* for each subscale was as follows: act with awareness (.81), observe (.78), describe (.85), nonjudging (.75), and nonreacting (.78).

The Emotion Regulation Questionnaire (Gross & John, 2003) examined emotion regulation strategies using two subscales: cognitive reappraisal (6 items) and expressive suppression (4 items). Respondents were asked to rate items using a 7‐point Likert scale ranging from 1 (*strongly disagree*) to 7 (*strongly agree*). The *α* for the present sample was .83 and .76 for cognitive reappraisal and expressive suppression, respectively.

The Perceived Stress Scale (PSS; Cohen et al., 1983) measures the extent to which respondents believe their lives have been unpredictable, uncontrollable, and overwhelming over the previous month. Although the original PSS includes 14 items, a shortened version was used in the present study, which consisted of four of the original items (PSS‐4; Warttig et al., 2013). Participants rated the items using a 5‐point Likert scale ranging from 0 (*never*) to 4 (*very often*). The *α* for the present sample was .74.

Intentions to persist in, versus drop out of, university were assessed by implementing the same method as that of Hardre and Reeve (2003). This was created by using the same two items implemented by Vallerand et al. (1997). For this study, “school” was replaced with “university” to establish a focus on higher education. Questions were answered using a 7‐point Likert scale ranging from 1 (*not at all*) to 7 (*very much*). The *α* for the present sample was .76.

Insomnia symptoms were assessed using the Sleep Condition Indicator (SCI; Espie et al., 2014), a clinical screening tool developed to appraise insomnia symptoms against the DSM‐5 criteria for Insomnia Disorder. The scale consists of eight items, each scored between 0 and 4, designed to examine insomnia symptomology during the last month. Specifically, questions pertain to sleep onset latency, awakenings during the night, perceived sleep quality, impairment to daytime functioning, and symptom persistence. Items are summed to create a total score between 0 and 32. Typically, lower scores indicate greater insomnia symptom severity. However, for greater clarity, the scoring was reversed such that higher scores indicated greater symptom severity. Internal consistency was high in the present sample (*α* = .89).

### Follow‐up assessment

Each measure (excluding the ANRA) was included at each additional time point (posttest = T2; 1‐week follow‐up = T3; and 4‐week follow‐up = T4).

### Intervention protocols

#### Attachment security priming

Participants completed a daily attachment security priming exercise over the course of 5 days. In total, three supraliminal priming exercises were used: an audio‐guided visualization (Stevenson et al., 2021), a text prompt writing task, and a picture prompt writing task (see Table 1 for details).

**TABLE 1:** Task protocols for experimental conditions.

Attachment security priming	Audio‐guided visualization: Participants were asked to visualize a close attachment figure, the relationship they share with them, and to visualize this individual in a situation of dealing with life's difficulties. Developed by Stevenson et al.,^22^ each audio recording was comparable in length, approximately 10 min long, and followed a similar temporal structure. After listening to the recording, participants answered the following open‐ended questions: What were you asked to visualize during the exercise? What was the scenario you were asked to visualize; how did the exercise end; what were you instructed to do? In addition, on a 5‐point scale between “extremely easy” and “extremely difficult,” participants indicated the extent to which they could visualize the content described. Picture prompt writing task: Each picture prompt comprised an individual alongside their attachment figure. The exercise was timed, whereby participants could move on to the next question after 5 min. Here, participants were asked to carefully view the displayed image and subsequently write about the content depicted. The specific prompt was as follows: “*Below is a photograph which we would like you to write about. Please look at it carefully before beginning the task. There are no right or wrong answers, just write whatever comes to mind. Try to be descriptive. You may wish to write about what you think is happening in this picture, who the individuals are or what the relationship is between them, how they are feeling, how the photo makes you feel, what are they saying to each other, or anything else you can think of to describe this photo. Please type your thoughts in the space provided. You will have 5 minutes to complete this task. If you finish before the 5 minutes are up, please continue to think about the photograph and write down anything else that comes to mind about the relationship*.” Text prompt writing task: The exercise was timed whereby participants could move on to the next question after 10 min. Participants were instructed to carefully read the following prompt and subsequently indicate their thoughts in a written format: “*Please think about a relationship you have had in which you found that it was relatively easy to get close to the other person and you felt comfortable depending on the other person. In this relationship, you didn’t often worry about being abandoned by the other person and you didn’t worry about the other person getting too close to you. Now take a moment to think about what it is like being in this relationship. What is it like being with this person who makes you feel safe and secure? You may want to remember a time when you were actually with this person. Try and get a visual image in your mind. What would he or she say to you? What would you say in return? How do you feel when you are with this person? How would you feel if they were here with you now?*”
Mindfulness practice	Audio‐guided visualization: Participants were asked to follow along a brief mindfulness meditation exercise focusing on breath and thoughts. Participants were instructed to focus on the physical sensations of breathing and an awareness of their thoughts. The recorded instructions for this exercise were adapted from the mindfulness meditation exercise used by Segal et al.^61^ in Mindfulness Based Cognitive Therapy. After listening to the recording, participants answered the following open‐ended questions: What were you asked to visualize during the exercise? What was the scenario you were asked to visualize; how did the exercise end; what were you instructed to do? In addition, on a 5‐point scale between “extremely easy” and “extremely difficult,” participants indicated the extent to which they could visualize the content described. Text prompt writing task: The exercise was timed whereby participants could move on to the next question after 10 min. Participants were instructed to carefully read the following prompt and subsequently indicate their thoughts in a written format: “*You will now complete an on‐line writing task. Again, we ask that you complete this somewhere you won't be disturbed. Write about a real (and, if possible, recent), event that you experienced as stressful, and that you have never disclosed to anyone, or that you have only minimally disclosed. As you write, try to make sense of this event by exploring your deepest thoughts and feelings about it. How did this event affect your life? What role does this event play in your life in the present time? How may this event impact your future? Open yourself up to experiencing your thoughts and feelings as they occur in the present moment. Describe what you are feeling in your body as you write (i.e., tension, tightness, heat, tingling), and where you feel it (i.e., hands, legs, stomach, feet, neck). It is important that you not judge your thoughts or your feelings, and that you allow them space. It is also important that you allow your words to relay your thoughts and feelings without judgment. If you find yourself feeling judgmental, notice this feeling, and then gently let it go. Simply accept whatever arises, observe it, and let it pass. Acknowledge your thoughts and feelings without evaluating them as positive or negative; accept them with openness, as they are. If you find your thoughts wandering, consciously bring your attention back to your body, and to the present moment. Please write without stopping until the time is up and the arrow appears at the bottom right of the page. Don’t worry about spelling, grammar, punctuation, or sentence structure. If you find yourself running out of things to write, repeat the last thing you wrote until you think of something new to write*.” Focus exercise: The exercise was timed whereby participants could move on to the next question after 5 min. In a quiet environment free from potential disturbance, participants were asked to: “*Choose any object nearby (a pencil, your computer mouse) and really focus on it for one minute. Pretend you’re seeing it for the first time. Pay close attention to its shape, texture, and construction. This can help you clear your mind and reconnect with those everyday objects that surround you*.” This protocol was again repeated, where participants were asked to focus on a different object.

#### Mindfulness practice

Participants completed a daily mindfulness exercise over the course of 5 days. Exercises were chosen to match the length and valence of the security priming methods. In order to mirror the attachment priming intervention, three exercises were used: an audio‐guided visualization (Stevenson et al., 2021), a text prompt writing task, and a focus exercise.

#### Waitlist control

Participants in the control condition were told that the study was at full capacity and given the option to join a waitlist. Following a 5‐day period, the participants completed the appropriate follow‐up measures and were informed of the deception. Participants were informed that the study was at full capacity, a limited form of deception allowed under APA and Helsinki guidelines deemed necessary for the study, posed minimal risk, and did not compromise participants' rights. In this waitlist‐controlled RCT, the approach reduced expectancy effects and did not alter consent or intervention access, and all participants were fully debriefed to safeguard trust.

### Procedure

Participants completed the battery of baseline measures at their own convenience. The experimental interventions (attachment security priming, mindfulness practice) were completed remotely via Qualtrics. Each intervention spanned five consecutive days where participants completed one experimental task per day. Depending on the task, the duration ranged between 5 and 10 min. Moreover, filler items were used that obscured the study aims. Immediately following their completion of the intervention, participants completed the same baseline battery of questionnaires. Next, participants were provided with a funneled debrief upon completion to evaluate awareness of the study design and aims. The questionnaire measures were repeated at T2, T3, and T4 before receiving a full study debrief. Participants allocated to the waitlist control condition were informed of the use of deception and that the study had reached full capacity and would be contacted to participate at a later date. This approach was used to minimize expectancy effects and demand characteristics, which may bias self‐report outcomes. Participants were not denied access to any mental health services and were free to seek support or withdraw participation throughout the study.

### Statistical analyses

All analyses were conducted using IBM SPSS v.27.0. Correlational analyses (Pearson's bivariate) examined possible relationships between all measures at baseline. Descriptive presentation of the data includes mean values and standard deviations. A series of Pearson's bivariate correlational analyses determined the relationships between adult attachment orientation, dispositional mindfulness, and mental health outcomes (ERQ, PSS, dropout intention, and SCI) at baseline. To examine the efficacy of the intervention conditions, a series of Bonferroni‐corrected 4 (time) × 3 (condition) repeated‐measures ANCOVAs were conducted for all study variables as dependent variables. Covariates included age, sex, and previous experience with mindfulness use. Following Mauchly's test of sphericity, where required, the Greenhouse–Geisser correction was applied to ANCOVA testing. In addition, the effect size of each interaction was determined using the partial eta squared (*η* ^2^). Here, *η* ^2^ = .01 indicates a small effect, *η* ^2^ = .06 indicates a medium effect size, and 14+ indicating a large effect. Simple effects analyses were undertaken to explore significant interactions where appropriate. Effect sizes for between‐group differences were estimated using Cohen's *d* and are reported with corresponding confidence intervals. In line with conventional benchmarks, values of 0.20, 0.50, and 0.80 were considered to represent small, moderate, and large effects, respectively. Significance was considered at the *p* < .05 level in all analyses. All pairwise comparisons were adjusted using the Bonferroni correction to control Type I error rates arising from multiple testing, consistent with established standards for rigorous statistical inference. No significant differences were reported between conditions for all baseline measures.

## RESULTS

Descriptive statistics and correlational outcomes for all measures (ECR‐12, ADA, FFMQ‐SF, ERQ, PSS, university dropout, and SCI) at each time point (T1–4) are reported in Tables 2 and 3, respectively.

**TABLE 2:** Means and standard deviations at four time points for three conditions.

Measures	Condition											
	Attachment (*n* = 41)				Mindfulness (*n* = 41)				Control (*n* = 40)			
	T1	T2	T3	T4	T1	T2	T3	T4	T1	T2	T3	T4
ECR‐12												
Anxiety	4.91 ± 1.24	4.54 ± 1.43	4.49 ± 1.29	4.41 ± 1.32	4.84 ± 1.28	4.67 ± 1.39	4.43 ± 1.46	4.38 ± 1.54	4.65 ± 1.64	4.69 ± 1.45	4.65 ± 1.49	4.65 ± 1.45
Avoidance	3.38 ± 1.30	3.20 ± 1.30	3.17 ± 1.24	3.09 ± 1.18	3.43 ± 1.24	3.52 ± 1.28	3.35 ± 1.23	3.24 ± 1.28	3.65 ± 1.52	3.76 ± 1.45	3.52 ± 1.29	3.58 ± 1.38
ADA	30.83 ± 11.26	29.07 ± 12.76	27.54 ± 12.00	27.10 ± 12.74	24.95 ± 9.95	24.56 ± 11.45	24.27 ± 12.29	23.27 ± 12.53	29.58 ± 13.00	29.48 ± 11.35	29.38 ± 12.17	29.48 ± 12.31
FFMQ												
Total	68.29 ± 11.77	71.02 ± 12.87	72.34 ± 11.90	73.05 ± 11.95	71.20 ± 13.16	69.54 ± 14.36	70.59 ± 12.57	73.39 ± 13.18	71.40 ± 12.20	70.02 ± 13.99	71.50 ± 11.94	70.97 ± 13.48
Act with awareness	14.27 ± 3.82	14.54 ± 4.51	15.29 ± 4.23	15.27 ± 4.41	15.00 ± 4.14	14.56 ± 4.18	14.54 ± 3.89	15.15 ± 4.41	15.40 ± 3.84	15.13 ± 4.36	14.95 ± 4.30	15.02 ± 4.29
Observe	13.17 ± 3.42	13.54 ± 4.00	13.41 ± 3.89	13.68 ± 4.02	13.05 ± 3.53	12.34 ± 3.43	12.54 ± 3.93	12.85 ± 4.42	13.20 ± 4.01	12.87 ± 4.07	13.40 ± 3.90	13.60 ± 3.56
Describe	15.59 ± 4.08	15.93 ± 3.72	16.66 ± 3.68	16.63 ± 3.79	16.27 ± 4.32	16.00 ± 4.17	16.05 ± 3.94	16.78 ± 3.92	14.75 ± 3.71	14.52 ± 4.08	15.08 ± 3.72	15.03 ± 4.18
Nonjudging	13.39 ± 3.90	13.68 ± 4.00	13.73 ± 3.72	13.24 ± 3.69	14.05 ± 3.85	12.90 ± 3.89	14.15 ± 4.26	14.34 ± 4.75	14.77 ± 4.39	14.45 ± 4.59	14.78 ± 4.13	14.13 ± 4.24
Nonreacting	11.88 ± 3.42	13.34 ± 3.34	13.24 ± 3.54	14.22 ± 3.04	12.83 ± 3.67	13.73 ± 4.14	13.32 ± 4.08	14.27 ± 3.89	13.27 ± 3.65	13.05 ± 4.33	13.30 ± 3.50	13.20 ± 4.08
ERQ												
Cognitive reappraisal	27.07 ± 5.48	28.29 ± 6.22	29.27 ± 5.34	29.56 ± 5.44	26.71 ± 7.39	27.17 ± 6.63	28.24 ± 6.11	29.12 ± 7.65	24.53 ± 7.43	26.63 ± 7.99	26.33 ± 7.89	26.10 ± 8.03
Expressive suppression	14.07 ± 5.25	13.44 ± 5.80	12.80 ± 5.01	13.15 ± 5.54	13.78 ± 5.70	13.61 ± 6.08	13.93 ± 6.21	13.27 ± 6.21	13.78 ± 5.69	14.95 ± 5.74	15.35 ± 5.77	14.93 ± 5.71
PSS	8.02 ± 2.95	8.07 ± 3.36	6.95 ± 3.12	6.83 ± 3.02	8.66 ± 2.30	8.59 ± 2.71	7.95 ± 2.63	7.27 ± 3.10	8.27 ± 3.03	7.80 ± 3.16	7.75 ± 3.23	8.05 ± 3.84
Dropout	6.85 ± 4.12	5.85 ± 3.66	5.56 ± 3.24	5.37 ± 3.06	5.76 ± 2.89	5.37 ± 2.55	5.15 ± 2.57	4.46 ± 2.12	5.75 ± 3.44	5.73 ± 3.52	5.20 ± 2.85	5.13 ± 2.91
SCI	14.66 ± 7.85	14.22 ± 8.18	13.51 ± 8.39	11.80 ± 8.67	12.37 ± 7.13	13.39 ± 7.46	11.59 ± 7.51	11.12 ± 7.54	12.70 ± 8.01	12.60 ± 7.66	12.30 ± 7.46	12.12 ± 7.97

**TABLE 3:** Descriptive statistics and correlation matrix of baseline measures (*N =* 122).

Measure	*M*	SD	1.	2.	3.	4.	5.	6.	7.	8.	9.	10.	11.	12.	13.
1. ECR‐12 anxiety	4.78	1.39													
2. ECR‐12 avoidance	3.48	1.35	.24** [.07, .41]												
3. Disorganized attachment	28.44	11.65	.23* [−.05, .39]	.27** [.10, .35]											
4. FFMQ‐SF total	70.29	12.37	−.62** [−.72, −.50]	−.37** [−.52, −.21]	−.27** [−.43, −.10]										
5. FFMQ‐SF act with awareness	14.89	3.93	−.46** [−.59, −.31]	−.30** [−.46, −.13]	−.33** [−.48, −.16]	.67** [.56, .76]									
6. FFMQ‐SF observe	13.14	3.63	−.16 [−.33, .02]	−.13 [−.30, .05]	.05 [−.13, .23]	.43** [.27, .56]	.07 [−.11, .25]								
7. FFMQ‐SF describe	15.54	4.06	−.43** [−.56, −.27]	−.46** [−.59, −.30]	−.17 [−.33, .01]	.74** [.64, .81]	.42** [.27, .56]	.17 [−.01, .34]							
8. FFMQ‐SF nonjudging	14.07	4.06	−.46** [−.59, −.31]	−.24** [−.40, −.07]	−.37** [−.51, −.20]	.69** [.58, .77]	.40** [.24, .54]	−.02 [−.20, .16]	.37** [.21, .52]						
9. FFMQ‐SF nonreacting	12.66	3.60	−.47** [−.60, −.32]	−.04 [−.21, .14]	−.03 [−.21, .15]	.67** [.56, .76]	.22* [.05, .39]	.22* [.04, .38]	.34** [.18, .49]	.40** [.24, .54]					
10. ERQ cognitive reappraisal	26.11	6.86	−.24** [−.40, −.07]	−.19* [−.36, −.01]	−.02 [−.20, .16]	.48** [.33, .60]	.20* [.02, .36]	.21* [.03, .37]	.40** [.24, .54]	.23* [.05, .39]	.50** [.36, .63]				
11. ERQ expressive suppression	14.34	5.53	.19* [.01, .35]	.68** [.57, .77]	.28** [.10, .43]	−.30** [−.45, −.13]	−.35** [−.49, −.18]	−.13 [−.30, .05]	−.33** [−.48, −.17]	−.23** [−.40, −.06]	.12 [−.06, .29]	−.10 [−.27, .08]			
12. Perceived stress	8.32	2.77	.58** [.45, .69]	.29** [.12, .44]	.14 [−.04, .31]	−.58** [−.68, −.44]	−.38** [−.53, −.22]	−.20* [−.36, −.02]	−.42** [−.56, −.26]	−.39** [−.53, −.23]	−.44** [−.57, −.28]	−.37** [−.51, −.20]	.19* [.02, .36]		
13. University dropout	6.12	3.53	.42** [.26, .56]	.09 [−.09, .27]	−.01 −.19, .17]	−.39** [−.53, −.23]	−.28** [−.44, −.11]	−.05 [−.23, .13]	−.33** [−.48, −.17]	−.32** [−.47, −.15]	−.25** [−.41, −.07]	−.19* [−.36, −.01]	.04 [−.14, .22]	.42** [.27, .56]	
14. SCI	13.76	7.68	.36** [.19, .50]	.27** [.10, .43]	.17 [.00, .34]	−.33** [−.48, −.16]	−.29** [−.44, −.12]	−.02 [−.19, .16]	−.33** [−.48, −.16]	−.16 [−.33, .01]	−.25** [−.41, −.08]	−.24** [−.40, −.07]	.18* [.01, .35]	.57** [.44, .68]	.35** [.19, .50]

*Note*: 95% Confidence intervals: [low, high].

* *p* < .05.

** *p* < .001.

### Primary outcomes

#### Baseline associations between adult attachment, mindfulness, and mental health

Attachment anxiety and avoidance were negatively related to total mindfulness and the facets of acting with awareness, describing, nonjudging, and nonreacting, whereas attachment disorganization was negatively related to total mindfulness, acting with awareness, and nonreacting. Both attachment anxiety and avoidance were significantly related to reduced cognitive reappraisal and increased reports of expressive suppression, perceived stress, university dropout, and insomnia severity. In contrast, total mindfulness was significantly related to increased reports of cognitive reappraisal and reduced reports of expressive suppression, perceived stress, university dropout, and insomnia severity.

#### Perceived stress

**FIGURE 2:**
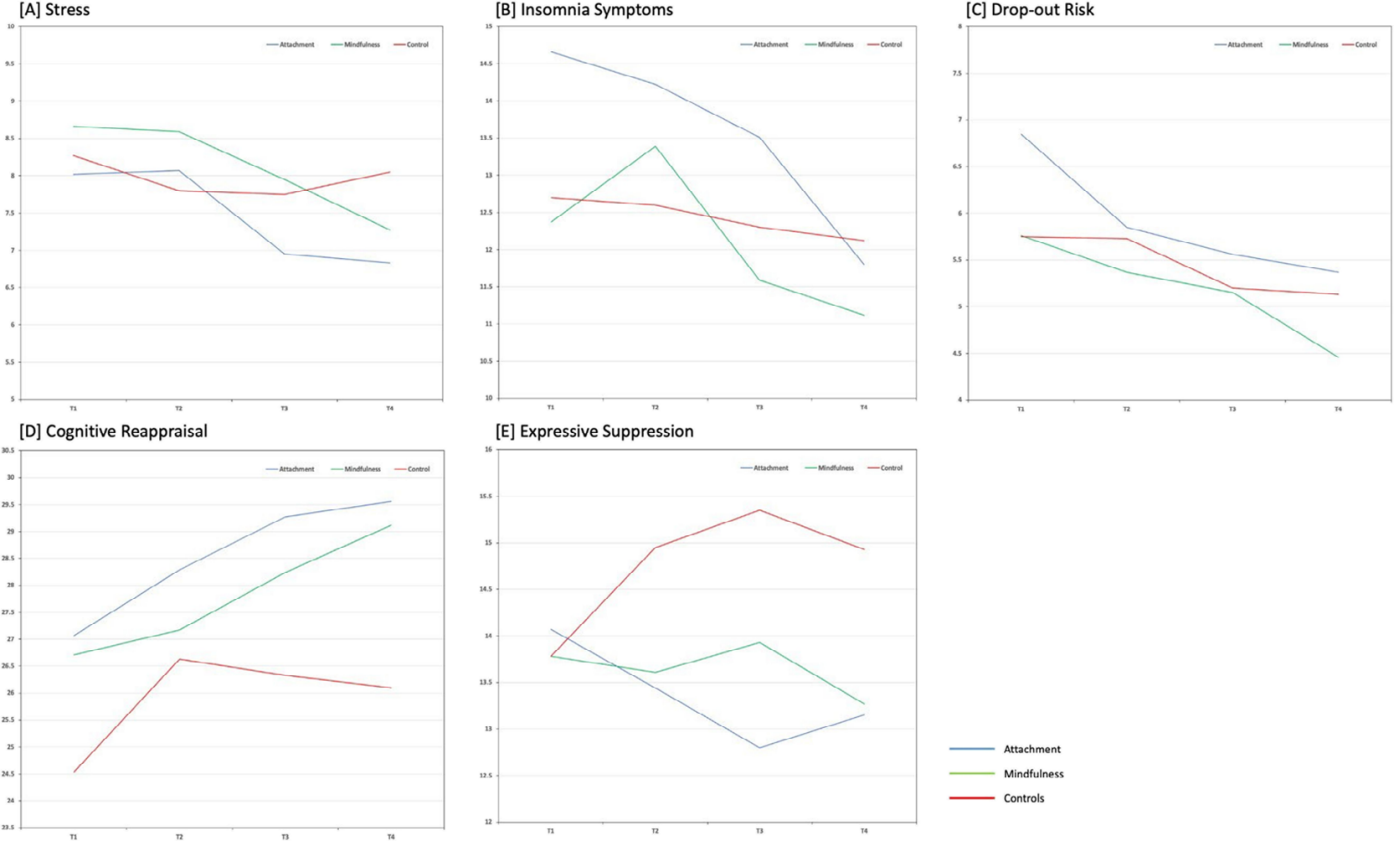
Mental health scores for each time point, split by condition.

Although no significant time × condition interaction was determined (*F* = 1.60, *p* = .15; *η* ^2^ = .03), a reduction in perceived stress was observed across time points in groups receiving intervention, whereas stress remained stable across time among waitlist controls (see Figure 2). Specifically, pairwise comparisons evidenced a significant reduction in perceived stress among those assigned to the attachment priming condition at T1 relative to T3 (*t*(40) = 2.59, *p* = .013, *d =* −.35, 95% CI [0.11, 1.53]), T1 relative to T4 (*t*(40) = 2.46, *p* = .018, *d* = −.40, 95% CI [0.21, 2.18]), T2 relative to T3 (*t*(40) = 3.25, *p* = .003, *d* = −.38, 95% CI [0.42, 1.82]), and T2 relative to T4 (*t*(40) = 3.10, *p* = .004, *d* = −.38, 95% CI [0.43, 2.05]). Likewise, significant reductions in perceived stress were reported among those assigned to the mindfulness practice condition at T1 relative to T4 (*t*(40) = 3.11, *p* = .003, *d* = −.51, 95% CI [0.49, 2.29]), T2 relative to T3 (*t*(40) = 2.70, *p* = .023, *d* = −.07, 95% CI [0.09, 1.18]), and T2 relative to T4 (*t*(40) = 3.12, *p* = .003, *d* = −.39, 95% CI [0.46, 2.17]). By the end of the interventional program (T4), students allocated to the attachment priming (6.83 ± 3.02) and mindfulness practice conditions (7.27 ± 3.10) displayed significantly reduced levels of perceived stress when compared with controls (8.05 ± 3.84). No significant main effects of time (*F* = 1.98, *p* = .077; *η* ^2^ = .02) or condition (*F* = 0.59, *p* = .556; *η* ^2^ = .01) were demonstrated.

#### Insomnia

No significant main effects of time (*F* = 0.42, *p* = .707; *η* ^2^ = .004) or condition (*F* = 0.53, *p* = .556; *η* ^2^ = .01) were determined. Although no significant time × condition interaction was determined (*F* = 1.79, *p* = .117; *η* ^2^ = .03), paired samples *t*‐tests determined significantly improved sleep among those assigned to receiving intervention, whereas reports of insomnia symptoms remained stable across time among waitlist controls. Specifically, pairwise comparisons evidenced a significant reduction in insomnia symptoms among those assigned to the attachment priming condition at T1 relative to T4 (*t*(40) = 3.79, *p* = .001, *d* = −.35, 95% CI = 1.33–4.38), T2 relative to T4 (*t*(40) = 4.41, *p* = .000, *d* = −.29, 95% CI [1.31,3.52]), and T3 relative to T4 (*t*(40) = 2.88, *p* = .006, *d* = −.02, 95% CI [0.51, 2.91]). Significant improvements of insomnia symptoms were also reported among those assigned to the mindfulness practice condition, although only at T2 relative to T3 (*t*(40) = 3.47, *p* = .001, *d* = −.24, 95% CI [0.75, 2.86]) and T2 relative to T4 (*t*(40) = 3.25, *p* = .002, *d* = −.30, 95% CI [0.86, 3.68]). By the end of the interventional program (T4), students allocated to the attachment priming (11.80 ± 8.67) and mindfulness practice conditions (11.12 ± 7.54) presented significantly reduced reports of insomnia symptoms when compared with controls (12.12 ± 7.97).

#### Emotion regulation

The results failed to evidence a significant condition × time interaction (*F* = 0.97, *p* = .494; *η* ^2^ = .01) when examining cognitive reappraisal. However, paired samples *t*‐tests determined that individuals subject to attachment priming presented a dose–response increase in cognitive reappraisal. Here, significant improvements were observed between T1 relative to T3 (*t*(40) = −2.70, *p* = .010, *d* = .41, 95% CI [−0.38, −0.55]), T1 relative to T4 (*t*(40) = −3.05, *p* = .004, *d* = .46, 95% CI [−4.14, −0.84]), and T2 relative to T4 (*t*(40) = −2.09, *p* = .045, *d* = .22, 95% CI [−2.51, −0.03]). A similar pattern emerged in those receiving mindfulness practice, where cognitive reappraisal significantly improved between T1 relative to T4 (*t*(40) = −2.46, *p =* .019, *d* = .22, 95% CI [−0.40, −0.43]). By the end of the interventional program (T4), students allocated to the attachment priming (29.56 ± 5.44) and mindfulness practice conditions (29.12 ± 7.65) displayed significantly greater levels of cognitive reappraisal when compared with controls (26.10 ± 8.03). No significant main effects of time (*F* = 0.76, *p* = .515; *η* ^2^ = .01) or condition (*F* = 1.56, *p* = .214; *η* ^2^ = .03) were observed when examining cognitive reappraisal.

The results demonstrated a significant condition × time interaction (*F* = 3.30, *p* = .027; *η* ^2^ = .05) when examining expressive suppression. However, paired samples *t*‐tests revealed a significant reduction in expressive suppression in those assigned to the attachment priming condition at T1 relative to T3 (*t*(40) = 3.20, *p* = .003, *d* = −.25, 95% CI [0.47, 2.07]) and T1 relative to T4 (*t*(40) = 3.20, *p* = .003, *d* = −.17, 95% CI [−0.16, 2.01]). By the end of the interventional program (T4), students allocated to the attachment priming (13.15 ± 5.54) and mindfulness practice conditions (13.27 ± 6.21) displayed significantly reduced levels of expressive suppression when compared with controls (14.93 ± 5.71). Although no main effect of condition (*F* = 0.32, *p* = .727; *η* ^2^ = .001) was observed, a significant main effect of time (*F* = 6.12, *p* = .008; *η* ^2^ = .05) was demonstrated.

#### University dropout

The results demonstrated reduced intention to dropout of university across time points among students receiving intervention, whereas intent to dropout remained stable across time among waitlist controls (*F* = 1.84, *p* = .120; *η* ^2^ = .03). Specifically, paired samples *t*‐tests determined significantly reduced reports of dropout intention in those assigned to the attachment priming condition at T1 relative to T2 (*t*(40) = 2.74, *p* = .009, *d* = −.26, 95% CI [0.26, 1.74]), T1 relative to T3 (*t*(40) = 3.42, *p* = .001, *d* = −.35, 95% CI [0.53, 2.06]), and T1 relative to T4 (*t*(40) = 3.31, *p* = .002, *d* = −.41, 95% CI [0.58, 2.40]). Likewise, significant reductions were also reported among those assigned to the mindfulness practice condition at T1 relative to T4 (*t*(40) = 3.24, *p* = .002, *d* = −.51, 95% CI [0.49, 2.10]) and T2 relative to T4 (*t*(40) = 4.51, *p* = .000, *d* = −.35, 95% CI [0.50, 1.31]). Participants assigned to the control condition also reported significant reductions in dropout intent at T2 relative to T3 (*t*(39) = 2.72, *p* = .010, *d* = −.17, 95% CI [0.14, 0.92]) and T2 relative to T4 (*t*(39) = 2.66, *p* = .011, *d* = −.19, 95% CI [0.14, 1.06]). By the end of the interventional program (T4), students allocated to the attachment priming (5.37 ± 3.06) and mindfulness practice conditions (4.46 ± 2.12) displayed significantly greater reductions in dropout intent compared with controls (5.13 ± 2.91). No significant main effects of time (*F* = 0.79, *p* = .502; *η* ^2^ = .01) or condition (*F* = 0.64, *p* = .527; *η* ^2^ = .01) were observed.

### Secondary outcomes

#### Attachment anxiety

**FIGURE 3:**
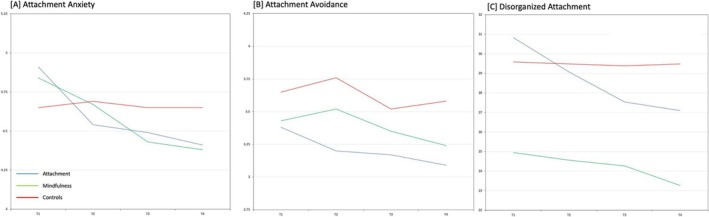
Attachment scores for each time point, split by condition.

The results demonstrated a significant time × condition (*F*(3, 348) = 4.81, *p* = .004, *η* ^2^ = 0.04) interaction, whereby pairwise comparisons indicated significant differences between T1 and T3 (*p* = .006, *d* = −.33) and T1 and T4 (*p* = .002, *d* = −.39: see Figure 3). On further examination, paired samples *t*‐tests determined a significant reduction in attachment anxiety in the attachment security priming and mindfulness practice conditions but not the control group at T3 relative to T1 (attachment priming: *t*(40) = 2.45, *p* = .019, *d* = .33, 95% CI [0.07, 0.75]; mindfulness practice: *t*(40) = 3.17, *p* = .003, *d* = .30, 95% CI [0.15–0.67]) and at T4 relative to T1 (attachment priming: *t*(40) = 3.32, *p* = .002, *d* = .39, 95% CI [0.19, 0.80]; mindfulness practice: *t*(40) = 3.05, *p* = .004, *d* = .33, 95% CI [0.15, 0.76]). However, no significant main effects of time, condition, or condition × time interaction were observed.

#### Attachment avoidance

Although no significant time × condition interaction was determined (*p* > .05), pairwise comparisons reported significant differences between T2 and T4 (*p* = .032, *d* = −.09). Specifically, paired samples *t*‐tests determined a significant reduction in attachment avoidance among those allocated to the mindfulness practice condition at T4 relative to T2 (*t*(40) = 2.71, *p* = .010, *d* = .22, 95% CI [0.07, 0.49]). However, no significant main effects of time and condition were observed.

#### Disorganized attachment

Although no significant time × condition interaction was determined (*p* > .05), pairwise comparisons reported significant differences between T1 and T4 (*p* = .034, *d* = −.31). Here, a dose–response reduction in disorganized attachment was observed in groups receiving intervention, whereas levels of disorganized attachment remained stable across time among waitlist controls. On further examination, paired samples *t*‐tests revealed a significant reduction in disorganized attachment from T1 relative to T3 (*t*(40) = 2.99, *p* = .005, *d* = .47, 95% CI [1.07, 5.50]) and T1 relative to T4 (*t*(40) = 2.84, *p* = .007, *d* = −.44, 95% CI [1.07, 6.39]) following attachment priming. No significant main effects of time and condition were observed.

#### Total mindfulness

**FIGURE 4:**
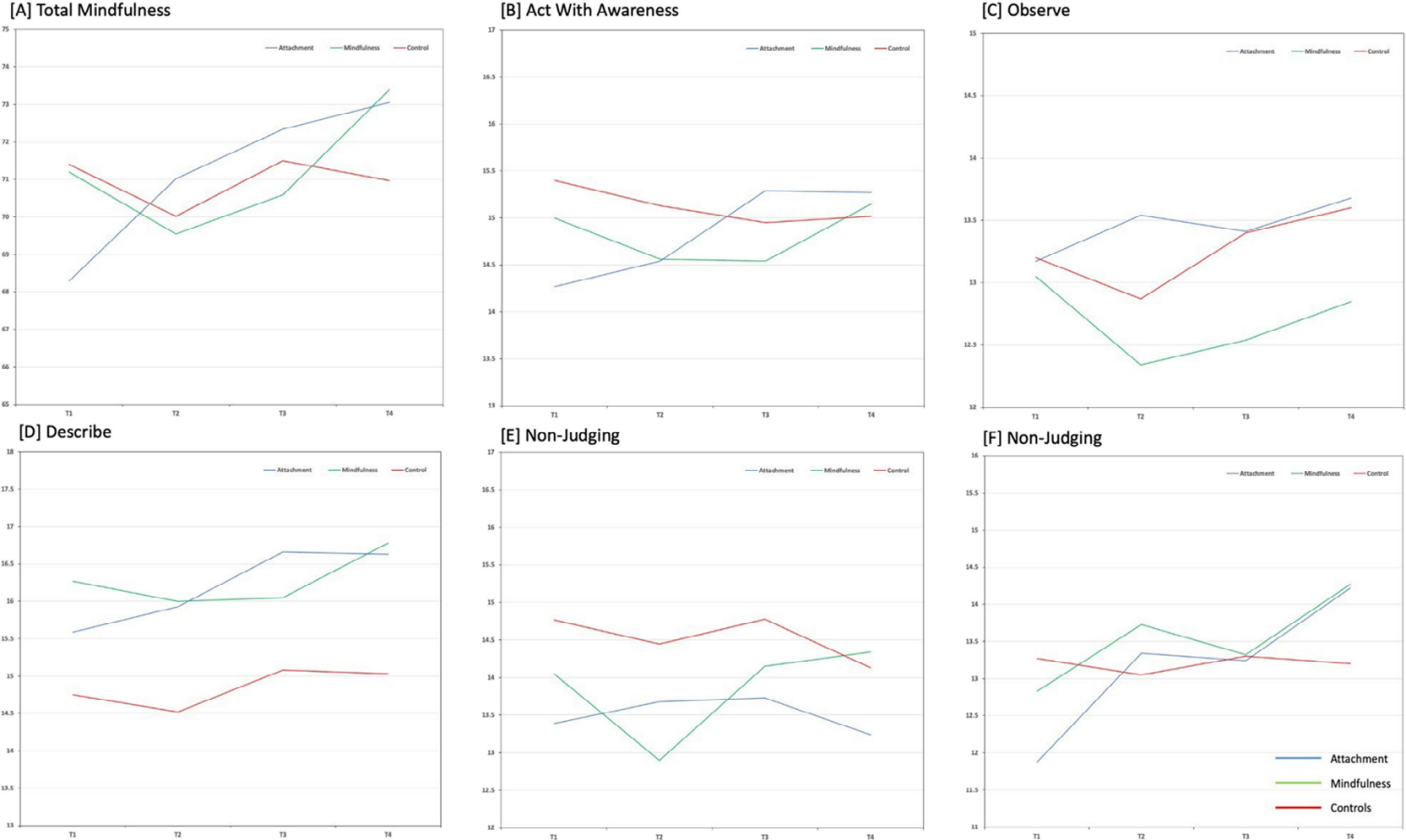
Mindfulness scores for each time point, split by condition.

The results demonstrated a significant interaction between time × condition (*F*[5.19,301.07] = 3.14, *p* = .008), revealing significant improvements in dispositional mindfulness among those receiving intervention relative to waitlist controls (see Figure 4). Pairwise comparisons reported significant differences between T1 and T4 (*p* = .045, *d* = .40) and T2 and T4 (*p* = .003, *d* = .16). Individuals subject to attachment priming presented a dose–response increase in dispositional mindfulness (see 4A). Here, paired samples *t*‐tests evidenced a significant increase in total mindfulness at T1 relative to T4 (*t*(40) = −3.47, *p* = .001, *d* = .40, 95% CI [−7.52, −1.99]) and at T2 relative to T4 (*t*(40) = −2.02, *p* = .050, *d* = .16, 95% CI [−4.05, 0.00]). Those receiving mindfulness practice demonstrated dose–response improvements from T2 following an initial reduction between T1 and T2. Specifically, total mindfulness significantly increased at T2 relative to T4 (*t*(40) = −3.18, *p* = .003, *d* = .28, 95% CI [−6.30, −1.41]). By the end of the intervention program (T4), students allocated to the attachment priming (73.05 ± 11.95) and mindfulness practice conditions (73.39 ± 13.18) displayed significantly greater levels of total mindfulness when compared with controls (70.97 ± 13.48). However, no significant main effect of time or condition was reported.

#### Act with awareness

The results failed to demonstrate a condition × time interaction. However, paired samples *t*‐tests determined that those receiving attachment priming demonstrated dose–response improvements across each time point. Here, improved awareness was observed between T1 relative to T3 (*t*(40) = −2.36, *p* = .023, *d* = .25), T2 relative to T3 (*t*(40) = −3.39, *p* = .002, *d* = .17), and T1 relative to T4 (*t*(40) = −2.35, *p* = .024, *d* = .24). By the end of the interventional program (T4) students allocated to the attachment priming (15.27 ± 4.41) but not mindfulness practice conditions (15.15 ± 4.41) displayed significantly greater levels of the act with awareness facet of mindfulness when compared with controls (15.02 ± 4.29). No significant main effects of time or condition were observed.

#### Observe

No significant condition × time interaction or main effects of time and condition were reported.

#### Describe

Although no condition × time interaction was reported, paired samples *t*‐tests determined a significant increase in describe among those allocated to the attachment priming condition at T1 relative to T3 (*t*(40) = −2.275, *p* = .028, *d* = .28, 95% CI [−2.03, −.12]) and T1 relative to T4 (*t*(40) = −2.31, *p* = .026, *d* = .26, 95% CI [−1.97, −.13]). By the end of the interventional program (T4), students allocated to the attachment priming (16.63 ± 3.79) but not mindfulness practice conditions (16.78 ± 3.92) displayed significantly greater levels of the describe facet of mindfulness when compared with controls (15.03 ± 4.18). No significant main effects of time or condition were reported.

#### Nonjudging

The results demonstrated a significant condition × time interaction (*F*(5.13, 348) = 2.34, *p* = .040), whereby improvements in nonjudging were observed in those receiving intervention relative to waitlist controls. Those receiving mindfulness practice demonstrated dose–response improvements from T2 following an initial reduction between T1 and T2 (*t*(40) = 2.47, *p* = .018, *d* = −.30, 95% CI [.21, −2.08]). Here, paired samples *t*‐tests determined an increase in nonjudging at T2 relative to T3 (*t*(40) = −2.93, *p* = .006, *d* = .31, 95% CI [−2.10, −.39]), T2 relative to T4 (*t*(40) = 2.95, *p* = .005, *d* = .35, 95% CI [−2.43, −0.45]). However, no significant main effects of condition or time were observed.

#### Nonreacting

The results demonstrated a significant main effect of time × condition (*F*(6, 348) = 2.80, *p* = .011, *η* ^2^ = .046), revealing significant improvements in nonreacting among those receiving intervention relative to waitlist controls. Paired samples *t*‐tests determined a significant increase in nonreacting among those allocated to attachment priming at T1 relative to T2 (*t*(40) = 3.66, *p* = .001, *d* = .31, 95% CI [−3.15, −.46]), T1 relative to T3 (*t*(40) = −2.59, *p* = .013, *d* = .39, 95% CI [−2.43, −.30]), T1 relative to T4 (*t*(40) = −4.92, *p* = .000, *d* = .72, 95% CI [−3.30, −1.38]), and T3 relative to T4 (*t*(40) = −2.63, *p* = 0.12, *d* = .30, 95% CI [−1.72, −.23]). Likewise, a dose–response increase in nonreacting was also observed in those allocated to the mindfulness practice condition at T1 relative to T2 (*t*(40) = −2.33, *p* = .025, *d* = .23, 95% CI [−1.30, −1.16]), T1 relative to T4 (*t*(40) = −3.19, *p* = .003, *d* = .38, 95% CI [−2.35, −.53]), and T3 relative to T4 (*t*(40) = −2.17, *p* = .036, *d* = .24, 95% CI [−1.84, −.06]). By the end of the interventional program (T4), students allocated to the attachment priming (14.22 ± 3.04) and mindfulness practice conditions (14.27 ± 3.89) displayed significantly greater levels of nonreactivity when compared with controls (13.20 ± 4.08). No significant main effects of condition or time were reported.

## DISCUSSION

The present study aimed to (i) examine and compare the efficacy of repeated mindfulness practice and attachment security priming on improving specific domains of student mental health and (ii) determine the directional influence and relationship between repeated security priming and trait mindfulness.

### Mindfulness and security priming in improving student sleep, stress, and emotion regulation

The outcomes indicate therapeutic effects of five consecutive digitally delivered sessions of attachment priming and mindfulness. Relative to waitlist controls, students receiving treatment displayed significant improvements in perceived stress, insomnia symptoms, emotion regulation, and dropout intention compared to baseline. Here, attachment priming yielded more promising short‐ and long‐term improvements in student mental health when compared with MBP. Deploying mindfulness nevertheless remains a favorable and generally beneficial approach to managing symptoms of insomnia and stress (Chen et al., 2020; Grossman et al., 2010). However, given the current outcomes, attachment priming offers a simple and effective alternative, particularly when the feasibility of mindfulness may be compromised. Involving three supraliminal exercises (audio‐guided visualization, text, and picture prompt writing task), vulnerable students may conceivably favor attachment priming when considering the creative nature of the exercises, reduced cognitive demand and time consumption relative to mindfulness, and minimal risk of adverse consequences.

University students remain consistently exposed to stressful experiences that contribute to psychological distress. Typically, university study involves increased standards and expectations whilst also encouraging greater academic independence (Cleary et al., 2010). Moreover, increased social activity in the absence of parental oversight provides opportunities for increased alcohol intake, possible experimentation of illegal substances, and formation of new friendship groups and sexual relationships (Vallerand et al., 1997). In the United Kingdom, the prospect of future debt, current financial difficulties, and the challenges associated with living on a self‐managed and reduced budget for the first time may present challenges for this population (Hardre & Reeve, 2003). This may be further accentuated among those struggling with the increasingly higher costs of living and increased tuition fees, particularly those relocating for study. Despite being the first point of referral, institutional well‐being services in the United Kingdom consistently fail to meet the demand of students seeking support for their mental health (see Gardani et al., 2022). Here, digital delivery of mindfulness practice and attachment priming may serve as an adjunct or standalone alternative for institutions failing to provide students with an adequate degree of support in a timely manner.

### Improving adult attachment and dispositional mindfulness

When concerning attachment security, both treatment approaches improved the established dimensions of attachment insecurity (anxiety and avoidance). However, only attachment priming demonstrated a therapeutic effect of improved attachment security by decreasing attachment disorganization. More crucially, the present study is the first to examine the potential therapeutic value of repeated attachment priming and mindfulness practice to diminish disorganized attachment. Although nascent relative to existing attachment theory, these findings widen the literature necessary to further delineate adult attachment disorganization. Here, this cost‐effective protocol may be extrapolated to additional nonclinical sample populations that remain understudied, serving to improve attachment security by reducing disorganization and its associated maladaptive outcomes (Paetzold et al., 2015).

In relation to total dispositional mindfulness, the current outcomes indicate therapeutic effects of each treatment approach, where total mindfulness scores demonstrated a significant increase following the treatment protocol when compared with controls. Exploring individual facets of mindfulness, when compared with controls, the repeated attachment priming improved: *act with awareness, describe, and nonreacting*, whereas repeated mindfulness practice yielded therapeutic effects for *act with awareness, describe, nonjudging, and nonreacting*.

### The relationship between adult attachment and mindfulness

Here, we further delineate the nature of the adult attachment and mindfulness relationship in the context of direction and causality. Although the relationship between these constructs is typically considered bidirectional in nature, we expand on previous research that provided preliminary evidence of attachment orientation being an antecedent in the development of mindfulness (Stevenson et al., 2021). As such, the present results support their theory that the relationship is not bidirectional as previously thought, but rather attachment orientation plays a causal role in the development of mindfulness. Furthermore, we also present the efficacy of repeated attachment priming in improving total mindfulness over and above that of mindfulness practice, whereas the reverse was not true.

If the relationship between attachment and mindfulness were bidirectional in nature, then it would have been appropriate to predict that the dose–response effect of both experimental conditions on both attachment and mindfulness outcomes would yield similar results. However, this is not the case, specifically when considering attachment avoidance, which increased following the mindfulness intervention. Additionally, in comparison, the immediate posttest dose–response effect of repeated attachment priming reduced all three dimensions of attachment insecurity. These findings further indicate the involvement of attachment anxiety as the mechanism by which individuals develop mindfulness and the capacity to be mindful and are thought to be a result of maladaptive emotion regulation abilities (Caldwell et al., 2010; Stevenson et al., 2021).

The efficacy of attachment priming, over and above mindfulness practice, may lie in the activation of internal working models and the ability to cognitively appraise representations of safety and support that facilitate affect regulation and adaptive functioning (see Grossman et al., 2010). Conversely to mindfulness, which requires sustained attentional training, attachment security priming may facilitate more immediate emotional benefits. There is also evidence that security priming can increase willingness to engage in mindfulness practice (Rowe et al., 2016). More broadly, the literature indicates that attachment security priming is associated with improved positive affect and reduced negative affect.^60^ The present research presents support for these findings while presenting the long‐term efficacy of attachment security priming, supporting its potential utility as a brief psychological intervention with sustained impact at follow‐up.

### Strengths and limitations

Several limitations of the present study should be considered. The population targeted was UK undergraduate students due to the increased prevalence of mental health difficulties within this population. As such, it is not possible to generalize the findings beyond this sample. Indeed, additional exploration in the general population should be considered moving forward. Moreover, the target sample size of the current trial was based upon a power calculation to ensure adequate ability to detect medium effect sizes. As the interventions were digitally delivered, attentional checks and daily email reminders ensured that participants completed the protocol as instructed.

The gender imbalance in our sample should be considered when interpreting these findings, as it may limit external validity and reduce confidence in the extent to which the results generalize across genders. Consistent with current guidance on the consideration of sex and gender in clinical research, we acknowledge this imbalance explicitly as a study limitation and interpret the findings with appropriate caution (Heidari et al., 2016; Nielsen et al., 2017). Future studies should recruit more gender‐balanced samples and include prespecified sex‐ or gender‐based analyses, with stratified randomization where appropriate, to assess the consistency of intervention effects across groups and strengthen external validity (Heidari et al., 2016; Nielsen et al., 2017). Replication across different educational settings and nonstudent populations will also be important to establish the robustness and wider applicability of these interventions.

### Practical implications

Research has documented the substantial need for mental health information and willingness to engage with digital tools as a method to support their well‐being (Montagni et al., 2020). Here, we present the scalability and low resource requirements of attachment security priming and mindfulness‐based exercises, making them suitable for incorporation into university mental health provision. Institutions could embed short digital practices, including guided visualizations and structured reflective prompts, within existing well‐being applications or online student platforms. Delivering these exercises through familiar digital systems would widen access, support early intervention, and ease pressure on counselling services by offering students timely and straightforward support options (Borghouts et al., 2021).

### Future directions

Future studies should compare digital and face‐to‐face delivery of attachment priming and mindfulness interventions to determine whether mode of delivery influences efficacy, engagement, and the persistence of effects. Face‐to‐face delivery may provide additional interpersonal and contextual benefits, whereas digital formats may offer greater accessibility and scalability. Trials in diverse student populations are needed to define the relative advantages of each approach and to inform their use within university mental health services (Borghouts et al., 2021; Davies et al., 2014; Harrer et al., 2019).

### Conclusion

Further examining the efficacy of positive psychology, digitally delivered sessions of repeated attachment priming and mindfulness proved therapeutically beneficial among undergraduate students. Relative to waitlist controls, students receiving treatment displayed significant improvements in perceived stress, insomnia symptoms, emotion regulation, and dropout intention compared with baseline. Each experimental protocol also led to differential improvements in facets of mindfulness and attachment. We also, for the first time, determined the directionality between adult attachment and dispositional mindfulness across multiple time points. Overall, the current outcomes add to the body of literature highlighting the need for improvements and novel therapeutic additions to current practices of counselling and student support services in higher education institutions.

## CONFLICT OF INTEREST STATEMENT

The authors declare no conflicts of interest.

## ETHICS STATEMENT

The authors assert that all procedures contributing to this work comply with the ethical standards of the relevant national and institutional committees on human experimentation and with the Helsinki Declaration of 1975, as revised in 2008. All procedures involving human subjects/patients were approved by the University of Lincoln's Research Ethics Committee (Project ID = 2934). This protocol was preregistered on the Open Science Framework database (DOI: DOI 10.17605/OSF.IO/VM9JC). Voluntary online informed consent was provided by all participants prior to data collection.

## DECLARATION OF INTERVENTION FIDELITY

All intervention arms were delivered using predefined protocols and standardised materials. Participants received only the intervention content allocated to their assigned condition, and delivery procedures were maintained consistently across assessment periods.

## Data Availability

The data that support the findings of this study are available from the corresponding author upon reasonable request.
